# Mutation screening of *NOS1AP *gene in a large sample of psychiatric patients and controls

**DOI:** 10.1186/1471-2350-11-108

**Published:** 2010-07-05

**Authors:** Richard Delorme, Catalina Betancur, Isabelle Scheid, Henrik Anckarsäter, Pauline Chaste, Stéphane Jamain, Franck Schuroff, Gudrun Nygren, Evelyn Herbrecht, Anne Dumaine, Marie Christine Mouren, Maria Råstam, Marion Leboyer, Christopher Gillberg, Thomas Bourgeron

**Affiliations:** 1 Human Genetics and Cognitive Functions, Institut Pasteur, Paris, France; 2 INSERM U995, Institut Mondor de Recherche Biomedicale, Psychiatric Genetics, Créteil, France; 3 APHP, Hôpital Robert Debré, Child and Adolescent Psychiatry, Paris, France; 4 Department of Child and Adolescent Psychiatry, Göteborg University, Göteborg, Sweden; 5 APHP, Groupe Hospitalier Henri Mondor - Albert Chenevier, Department of Psychiatry, Créteil, France; 6 Saint George's Hospital Medical School, London, UK; 7 Université Denis Diderot Paris 7, Paris, France; 8 INSERM, U952, Paris, France; 9 UPMC Univ Paris 06, Paris, France

## Abstract

**Background:**

The gene encoding carboxyl-terminal PDZ ligand of neuronal nitric oxide synthase (*NOS1AP*) is located on chromosome 1q23.3, a candidate region for schizophrenia, autism spectrum disorders (ASD) and obsessive-compulsive disorder (OCD). Previous genetic and functional studies explored the role of *NOS1AP *in these psychiatric conditions, but only a limited number explored the sequence variability of *NOS1AP*.

**Methods:**

We analyzed the coding sequence of *NOS1AP *in a large population (n = 280), including patients with schizophrenia (n = 72), ASD (n = 81) or OCD (n = 34), and in healthy volunteers controlled for the absence of personal or familial history of psychiatric disorders (n = 93).

**Results:**

Two non-synonymous variations, V37I and D423N were identified in two families, one with two siblings with OCD and the other with two brothers with ASD. These rare variations apparently segregate with the presence of psychiatric conditions.

**Conclusions:**

Coding variations of *NOS1AP *are relatively rare in patients and controls. Nevertheless, we report the first non-synonymous variations within the human *NOS1AP *gene that warrant further genetic and functional investigations to ascertain their roles in the susceptibility to psychiatric disorders.

## Background

The current concepts adopted by DSM-IV and ICD-10 that define a psychiatric disorder as a single entity may be misleading and hamper the identification of susceptibility genes. Some disorders classically considered as separate conditions could be envisaged as being the phenotypic extremes of a continuum caused by an overlapping set of genes. Indeed, the same region on chromosome 1q23.3 was detected in whole genome scans for separate psychiatric disorders and could therefore contain one shared susceptibility gene for these conditions. For schizophrenia, several linkage studies detected a significant linkage at 1q23.3 [1-6]. The same region was detected in two linkage studies in autism spectrum disorders (ASD). Buxbaum et al., [7] reported an association between patients with autism and obsessive-compulsive behaviors (OCD), and D1S1677 (p value = 0.003 under multipoint nonparametric analyses). These results were replicated by Ylisaukko-oja et al., [8] who found a significant linkage between patients with Asperger syndrome, known to have frequent OC symptoms [9], and 1q23.3 (D1S484, LOD score = 3.58 under dominant model). In OCD patients a linkage at chromosome 1q23.3 was observed in the early-onset group (age at onset <18 years) [10]. Non parametric multipoint linkage analysis produced a maximum LOD score of 2.94 at D1S1679 with an empirical p value equal at 0.001 after permutations.

Several genes located in region 1q23.3 have been studied, e.g. the regulator of G-protein signalling 4 gene [11-14], the myelin protein zero like 1 gene [15], the *UHMK1 *gene [16], encoding for a serine/threonine protein kinase, and the carboxyl-terminal PDZ ligand of neural nitric synthase (*NOS1AP*) gene. Among these genes, *NOS1AP *remains the most promising candidate. Brzustowicz et al., [17] analyzed 14 microsatellites and 15 single nucleotide polymorphisms (SNPs) between D1S1653 and D1S1679, and found a significant association between *NOS1AP *and SCZ. These results were reproduced in three independent samples of patients [18-20]. NOS1AP is in adaptor protein, which binds to neuronal nitric oxyde synthase, eliciting S-nitrosylation and activation of Dextras 1, which regulate NMDA receptor-mediated glutamate neurotransmission [21]. Using quantitative real time polymerase chain reaction, Xu et al., [22] reported an increased of the NOS1AP short isoform mRNA in the dorso-prefrontal cortex of schizophrenic patients. The over expression this short isoform mRNA could disrupt NMDA receptor function [23] and increase the risk of a psychiatric condition such as SCZ, ASD or OCD. More recently, Wratten et al., [24] reported that a risk allele, located in intron 2 and associated with schizophrenia, could enhance transcription factor binding and increase gene expression. Together, these findings make *NOS1AP *as a compelling candidate for several psychiatric disorders. However, no coding mutation in *NOS1AP *has been reported [17] and the positive association only concerns SNPs that have no established functional role. The aim of this study was to explore the expression pattern of *NOS1AP *in human tissues and its sequence variability in a large population of subjects (n = 280), including patients with schizophrenia (n = 72), ASD (n = 81) or OCD (n = 34), and in healthy volunteers controlled for the absence of personal or familial history of psychiatric disorders (n = 93).

## Methods

### Patients

Schizophrenic (46 males, 26 females) and OCD (21 males, 13 females), fulfilling the DSM-IV criteria for their disorders, were recruited in Paris (Department of Adult Psychiatry, H. Mondor and A. Chenevier Hospitals, Créteil, and Department of Child and Adolescent Psychiatry, R Debré Hospital, France). Lifetime psychiatric evaluation was carried out during a direct interview by trained psychiatrists using the Diagnostic Interview for Genetic Studies (DIGS) [25] and the Kiddie-Schedule of Affective Disorders and Schizophrenia - epidemiologic version [26] for OCD probands aged under 18 years. Probands with ASD (72 males, 9 females; 46 trios, 35 multiplex families) were recruited by the Paris Autism Research International Sib-pair (PARIS) study. Patients with autism or Asperger's syndrome fulfilled the DSM-IV criteria for autistic disorder and the Autism Diagnostic Interview Revised algorithm for ICD-10 childhood autism and the Autism Diagnostic Interview-Revised [27] criteria for childhood autism. Subjects were included only after a thorough clinical and medical work-up comprising a full medical and family history, physical and neuropsychological examination, standard karyotyping and fragile-X testing, blood and urine analyses for metabolic screening. Healthy controls of European descent (n = 93) were recruited among blood donors at the Pitié-Salpétrière Hospital in Paris, France (49 males, 44 females). All controls were included after being interviewed with the DIGS and with the Family Interview for Genetic Studies [28] to confirm the absence of both personal and familial history of psychiatric disorders.

The local Research Ethics Boards reviewed and approved the study. Written informed consent was obtained from all probands and controls. If the proband was under 18 years old, the proband's consent and written parental consent were obtained.

### Gene characterization

The genomic structure of *NOS1AP *was obtained from http://genome.ucsc.edu/. Exon-intron boundaries were identified and primers designed to cover the regulatory splice site regions and the exons using the previous Web site. *NOS1AP *covers a genomic region of 298 Kb and comprises 10 exons. As reported by Xu et al., [19], the long isoform is composed of exons 1 to 10 and encodes a protein of 75 kDa. The short isoform is composed of exons 9 and 10, and encodes a protein of 30 kDa. Both proteins contain a PDZ domain.

### Expression of NOS1AP long and short isoforms in human tissues

One microgram of total RNA from different tissues and specifically from regions of the human brain was reverse transcribed using the Gene Amp RNA PCR kit (Perkin-Elmer Corp., Norwalk, CT) and primers located in exons 1-7, 6-9 and 8-10 (forward -reverse) for the long isoform and in the 3'UTR-exon10 for the short isoform [22]. The ages of the two males and the two females studied were 74, 42, 55, and 36 years-old, with a post-mortem delay of 10, 21, 24, and 2 h, respectively. Normal control human brains were obtained at autopsy under guidelines approved by the ethics committee

### Mutation screening in psychiatric patients

The mutation screening was conducted in two steps. First, the exonic sequences of *NOS1AP *were screened for genetic variants by direct sequencing with the BigDye kit v3.0 (Applied Biosystems) according to the manufacturer's instructions in a sample of schizophrenic patients (n = 72), ASD patients (n = 81) and healthy volunteers (n = 93). The exonic sequence and the adjacent intronic sequences were considered first as most likely to harbour functionally important variants. Primers were designed using Amplify V3.1 software http://engels.genetics.wisc.edu/amplify/ and are available on request. Then, the rare variants identified were screened by direct sequencing in a additionnal sample containing 224 patients with ASD and 225 patients with schizophrenia. Patients included in this second step fulfilled the same criteria as patients enrolled in the first steps of the study and described previously.

### Association study in schizophrenic and ASD patients

The association study was conduct using 11 SNPs with a minor allele frequency higher than 0.05 and identified by direct sequencing in the schizophrenic and ASD patients (table 1). The program HaploView (v3.32) was used to test for concordance with Hardy-Weinberg equilibrium (HWE), calculate D' between SNP pairs and identify linkage disequilibrium (LD) blocks, which were defined with confidence intervals [29]. The P values for each marker and haplotype were adjusted from permutation test with 10,000 simulations if significance was under 0.01. Comparison of genotype frequencies between cases and controls was performed with chi-square test. The association study was conducted in two steps: first, all the schizophrenic patients (n = 72) and all the ASD patients (n = 81) were compared to the healthy volunteers (n = 93) to detect a potential association. Second, we included additional schizophrenic patients (n = 224), ASD patients (n = 225) and controls (n = 193) to increase the power of detecting an association with the SNP rs348624 (R334R) located in exon 9. This SNP was previously reported to be associated with schizophrenia [19]. For the additional subjects, genotypes were determined by TaqMan assay for allelic discrimination using the ABI Prism^® ^7000 Sequence Detection System (Applied Biosystems, Foster City, CA). Schizophrenic and ASD patients were the same as those included in the second step of the mutation screening.

**Table 1:** Allele and genotype frequencies of frequent polymorphisms in NOS1AP*

**Polymorphisms**^**#**^	Location	Number of alleles (frequency)			Number of Genotype (frequency)			
		Allele 1	Allele 2	*P *(df = 1)	11	12	22	*P *(df = 2)
**rs10918776**	**Intron 2**	**G**	**C**					
SCZ		100 (0.77)	32 (0.23)	0.46	42	22	5	0.57^§^
ASD		117 (0.72)	45 (0.28)	0.06	39	37	4	0.06^§^
Controls		142 (0.80)	34 (0.20)		57	28	3	
**rs10800405**	**Intron 3**	**C**	**G**					
SCZ		91 (0.76)	31 (024)	0.92	37	25	3	0.98^§^
ASD		102 (0.74)	36 (0.26)	0.63	34	33	1	0.60^§^
Controls		113 (0.77)	35 (0.23)		43	29	3	
**rs347306 and rs347307**	**Intron 5**	**C and T**	**T and C**					
SCZ		74 (0.56)	60 (0.44)	0.19	20	38	12	0.23
ASD		82 (0.52)	76 (0.48)	0.98	17	41	20	0.46
Controls		87 (0.52)	81 (0.48)		15	54	18	
**rs3751285**	**Intron 6**	**A**	**G**					
SCZ		68 (0.77)	20 (0.33)	0.35	28	12	4	0.32^§^
ASD		97 (0.71)	39 (0.29)	0.96	34	27	6	0.97
Controls		106 (0.72)	42 (0.28)		39	31	6	
**rs3751284**	**Exon 6**	**C**	**T**					
SCZ		58 (0.66)	30 (0.34)	0.35	20	18	6	0.45
ASD		75 (0.55)	61 (0.45)	0.32	20	34	13	0.69
Controls		88 (0.60)	60 (0.40)		26	39	11	
**rs905721**	**Intron 8**	**C**	**T**					
SCZ		96 (0.70)	42 (0.30)	0.10	33	30	6	0.22
All SCZ		353 (0.62)	213 (0.38)	0.92	114	125	44	0.86
ASD		104 (0.66)	54 (0.34)	0.33	31	41	6	**0.02**
All ASD		360 (0.64)	198 (0.36)	0.41	115	130	34	0.27
Controls		98 (0.65)	52 (0.35)		37	24	14	
All controls		309 (0.62)	189 (0.38)		102	105	42	
**rs348624 and rs1964052**	**Exon 9 and intron 9**	**C and C**	**T and T**					
SCZ		121 (0.89)	15 (0.11)	0.48	54	13	1	0.49^§^
All SCZ		500 (0.87)	74 (0.13)	0.34	218	63	6	0.50
ASD		132 (0.85)	24 (0.15)	0.07	55	18	3	0.07^§^
All ASD		472 (0.86)	74 (0.14)	0.22	206	60	7	0.39
Controls		137 (0.91)	13 (0.09)		62	13	0	
All controls		283 (0.89)	34 (0.11)		128	29	2	
**rs164146 and rs164147**	**Exon 10**	**G and C**	**C and A**					
SCZ		104 (0.83)	22 (0.17)	0.71	43	18	2	0.73^§^
ASD		133 (0.84)	25 (0.16)	0.99	56	20	2	0.91^§^
Controls		128 (0.84)	24 (0.16)		57	23	1	

*Polymorphisms included have a minor allele frequency above 0.05. ^# ^Markers are listed from centromere au telomere. ^§ ^Fischer exact Test. Significant *P *values (<0.05) are in bold. Abbreviations: ASD: autism spectrum disorders, SCZ: schizophrenia.

## Results

### Expression of the NOS1AP long and short isoforms in the human tissues

**Figure 1:**
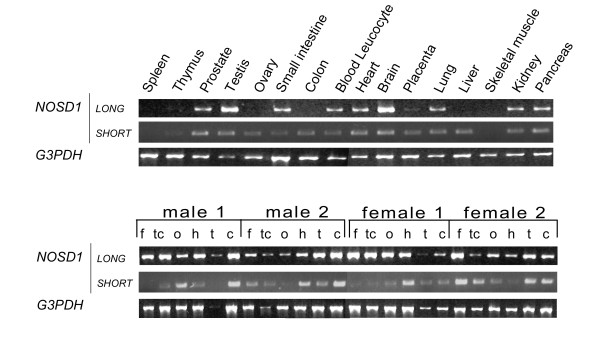
**Expression analysis of *NOS1AP *short and long isoforms in normal control human tissues**. One microgram of total RNA from different tissues and specifically from regions of the human brain was reverse transcribed using the Gene Amp RNA PCR kit (Perkin-Elmer Corp., Norwalk, CT) and primers located in exons 1-7, 6-9 and 8-10 (forward -reverse). The ages of the two males and the two females studied were 74, 42, 55, and 36 years-old, with a post-mortem delay of 10, 21, 24, and 2 h, respectively. f: frontal cortex, tc: temporal cortex, o: occipital cortex, h: hippocampus, t: thalamus; c: cerebellum. Normal control human brains were obtained at autopsy under guidelines approved by the ethics committee.

In order to study the expression pattern of the *NOS1AP *long and short isoforms in human tissues, we performed reverse transcriptase - polymerase chain reactions (RT-PCR) on RNA isolated from different tissues and brains regions (figure 1). Brain samples were obtained from four control individuals. These RT-PCR results are non quantitative and should be taken with care. However, *NOS1AP *long and short isoforms were not restricted to the brain, but were also detected in testis, prostate, small intestine, blood leukocyte, heart, placenta, lung, kidney and pancreas. In brain, the two isoforms of *NOS1AP *were also not restricted to a specific brain region (figure 1) and displayed high inter-individual differences.

### Mutation screening of NOS1AP in individuals with psychiatric disorders

**Figure 2:**
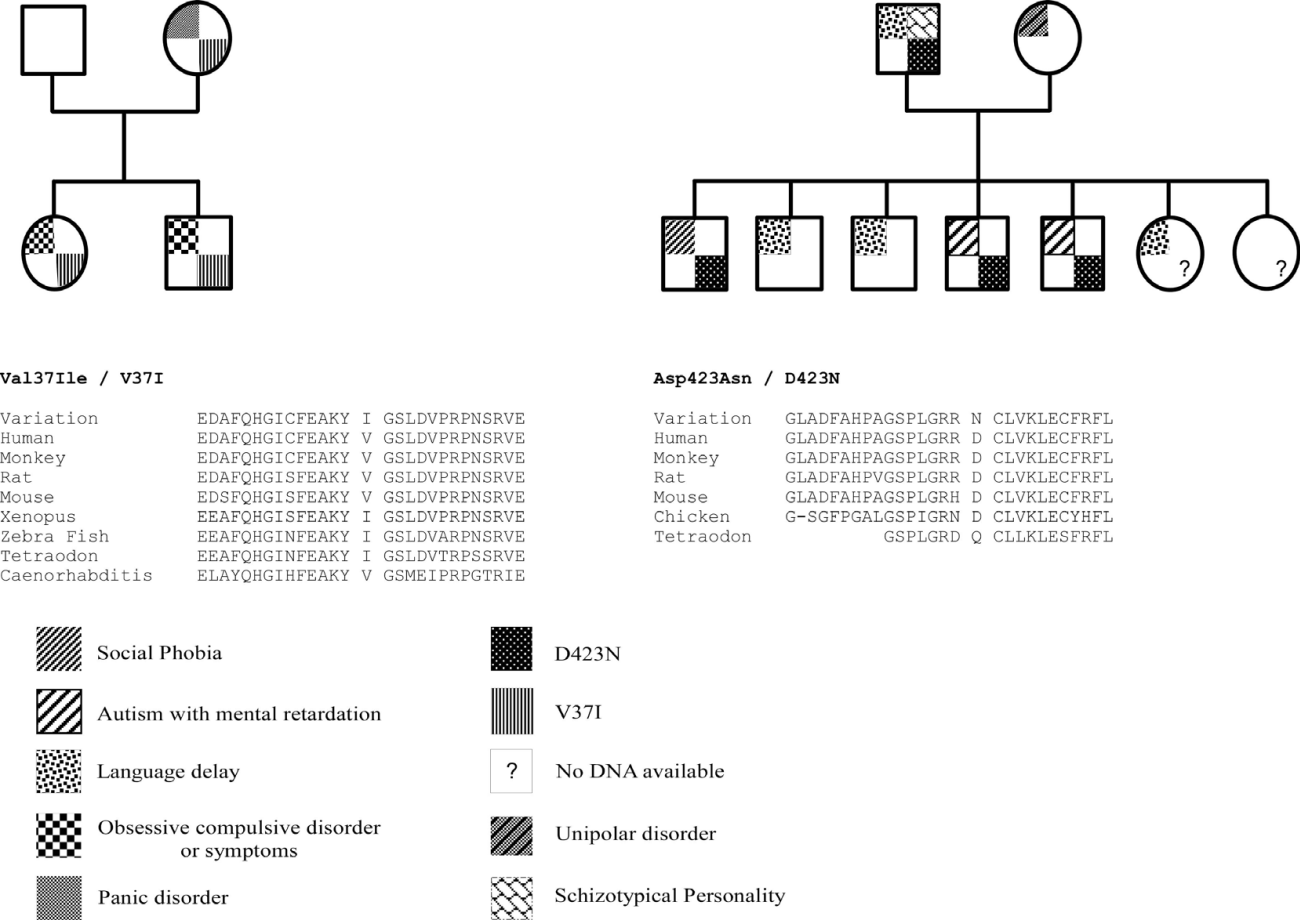
**Familial segregation and conservation among species of V37I and D423N located in *NOS1AP *gene**.

All the exons of *NOS1AP *were directly sequenced in a large subset of subjects (n = 280), including patients with schizophrenia (n = 72), ASD (n = 81) and OCD (n = 34), and in healthy volunteers (n = 93). Only two non-synonymous variations, V37I and D423N were identified (figure 2). V37I was detected in one female patient with OCD. The mutation was also present in her affected brother with OCD and was transmitted by the mother suffering from panic attack (figure 2). We were unable to detect this variation in the extended sample of patients with ASD (n = 224) or schizophrenia (n = 225). This rare variant was located in the phosphotyrosin-binding domain that is conserved among mammals, but not in Xenopus and Zebra fish, the latter having a valine to an isoleucine substitution.

The second non-synonymous variation, D423N, was detected in three brothers (figure 2), two affected with ASD and mild mental retardation, and one with social phobia. Two additional brothers with language delay and no autistic symptoms do not carry the mutation. The mutation was transmitted by the father who has a schizotypical personality and a history of language delay during childhood (figure 2). The variation D423N was absent from the extended sample of patients with ASD (n = 224), schizophrenia (n = 225) and an additional set of 95 controls from North African descent since proband had North African ancestors. D423 is highly conserved among species (figure 2) and is adjacent to the PDZ domain of the short and long isoform of the protein. The variation D423N changes a negatively charged amino acid into a polar uncharged residue. *In silico*, the variation D423N is predicted to alter the protein stability (delta G of -1.16) and function (SIFT program: score <0.001).

### Association study in schizophrenic and ASD patients

**Figure 3:**
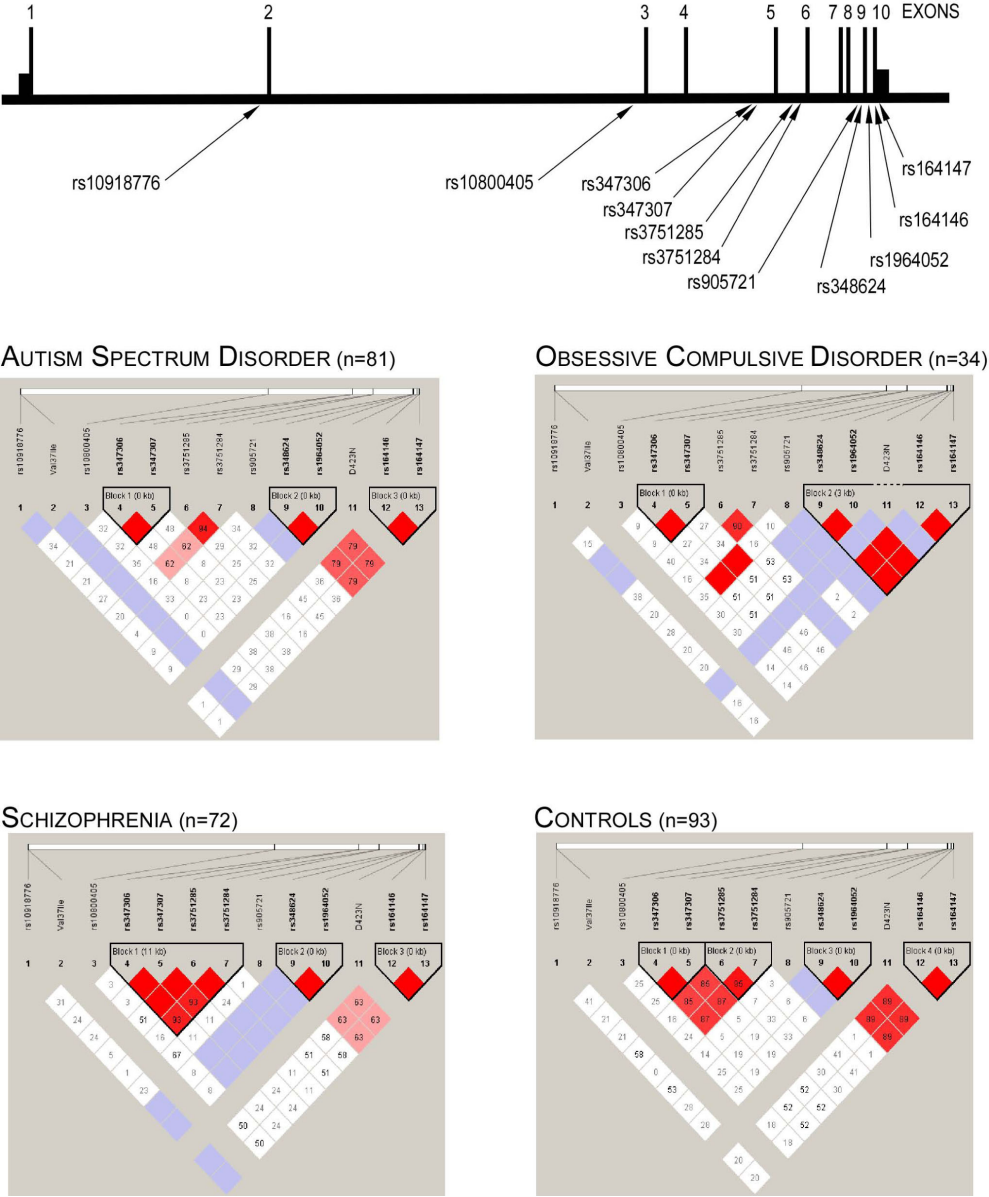
**Schematic representation of *NOS1AP *with location of frequent single nucleotide polymorphisms and Haploview-generated linkage disequilibrium map of the *NOS1AP***. Region of high LD (D' = 1) are shown in bright red. Only ASD, OCD, schizophrenic and control subjets patients are shown in the LD diagram. The numbers in the squares are D prime values (×100).

In order to test if frequent polymorphisms within *NOS1AP *were associated with susceptibility to psychiatric disorders, we assessed the frequency of 11 SNPs in patients and controls. Distributions of allelic frequencies of all markers were in Hardy Weinberg Equilibrium in cases and controls (p > 0.10). The rs347306 and rs347307 located in intron 5 are in strong LD whatever the population considered. Also, rs348624, rs1964052, rs164146 and rs164147 were in the same LD block located in the 3'region of *NOS1AP *(figure 3). We could not detect any significant allelic frequency difference between SCZ and controls or between ASD and controls (table 1). Haplotype analyses defined with confidence intervals did not find any significantly association either (table 2). In order to test the previously observed association between SCZ and SNP rs348624 (R334R) located in exon 9, an additional sample of SCZ (n = 224), ASD patients (n = 225) and controls (n = 193) were genotyped. However, no allelic association was observed.

**Table 2:** Estimated haplotype frequencies in *NOS1AP*

Haplotype block	Frequency (%)			*P *values	
	
	SCZ	ASD	Control	SCZ vs. control	ASD vs. control
1 (rs10918776- rs10800405)					
CT	0.55	0.48	0.48	0.23	0.98
TC	0.45	0.52	0.52	0.23	0.98
2 (rs3751285 - rs3751285)					
AG	0.56	0.46	0.59	0.28	0.45
GT	0.23	0.28	0.28	0.40	0.96
AT	0.11	0.17	0.13	0.74	0.33
3 (rs348624 - rs1964052)					
CC	0.89	0.85	0.91	0.50	0.07*
TT	0.11	0.15	0.08	0.50	0.07*
4 (rs164146 - rs164147)					
GC	0.83	0.84	0.84	0.71	0.99
CA	0.18	0.16	0.16	0.71	0.99

* *P *= 0.53 when 10000 permutations where simulated. Significant *P *values (<0.05) are in bold. Abbreviations: ASD: autism spectrum disorders, SCZ: schizophrenia.

## Discussion

The lack of association between frequent *NOS1AP *polymorphism and susceptibility to SCZ are in accordance with those obtained in a Caucasian population [30], but contrast with a case control study conducted in a Chinese population, reporting an association between SCZ and rs348624. These contradictory results could be due to a difference in allelic frequency between populations. Indeed, the major allele frequency for rs348624 in Chinese SCZ patients (0.87) is similar in our patients (SCZ 0.87; ASD 0.86), but the two control populations differ in allelic frequencies (Chinese controls: 0.82; French controls: 0.89). In addition, the association was only suggestive since the same group did not replicate their results when using a family-based approach [31].

The lack of association between frequent SNPs and the disorder does not exclude *NOS1AP *as a susceptibility gene to psychiatric conditions. Indeed, a larger sample size might detect an association between SCZ and frequent *NOS1AP *polymorphism. In addition, private mutations altering the function of NOS1AP may also contribute to the disorder in a subset of patients. Our mutation screening (280 subjects, including patients with SCZ, ASD, OCD, and controls) showed that variations altering the protein sequence of *NOS1AP *are rare - at least among individuals of European descent. Nevertheless, two changes V37I and D423N were identified in one family with OCD and in one family with ASD, respectively. In public databases three non synonymous variations for *NOS1AP*, namely: rs41405649, rs4127196, rs34398505 are reported http://www.ncbi.nlm.nih.gov. However, their genotype has not been validated by an independent method and their frequency has not been reported, except for rs34398505 (0.061). The single individual carrying this non synonymous rare variant (NA14649) is a female from African-American ancestor. The variation 37I reported in the OCD family, was present in two affected siblings and in the mother. The mother was directly interviewed, and she suffers from sever panic disorder. In the ASD family, the variation 423N segregated with the presence of psychiatric conditions related the broad autism phenotype [32,33]. The variant was present in the two affected male siblings, in the brother suffering from a social phobia and transmitted by the father who had a schizotypical personality. The two unaffected brothers do not carry the mutation and have no autistic symptoms. If *NOS1AP *plays a role in the susceptibility to ASD, it may represent only one of the contributing factors required for the full phenotypic trait of ASD. The conservation of 37I and 423N observed among species suggests that substitution could influence protein function. The mutation V37I only concerns the long *NOS1AP *isoform, whereas D423N is present in both the short and the long isoform of *NOS1AP*. Specifically, D423N mutation seems to decrease the protein stability as predicted by the Prediction of Protein Stability Changes upon Mutations Program http://www.ics.uci.edu/~baldig/mutation.html with a delta G of -1.16. Also, similar results were obtained using the SIFT program. The D to N substitution in position 423 is predicted to affect protein function http://sift.jcvi.org/ with a score under 0.001. At this stage, we lack information on the specific impact of these variants on NOS1AP function to decipher whether these mutations contribute or not to the disorder. Thus, the functional analyses of NOS1AP variants as well as the identification of the genetic/environmental factors, that modulate the expression of NOS1AP in the human brain, are needed to reinforce the potential implication of this gene in the susceptibility to OCD or ASD.

In our four controls individuals, expression of both NOSAP1 isoforms displayed high inter-individual differences whatever the explored brain regions. This inter-individual variability in the expression of NOS1AP might contribute to the increased of the short isoform mRNA in the dorso-prefrontal cortex of patients with SCZ [22].

## Conclusions

As reported by Brzustowicz et al., for SCZ [17], variations within the coding sequence of *NOS1AP *are rare and therefore cannot be considered as susceptibility factors to ASD, OCD and SCZ. However, we cannot exclude that for a very small subset of patients with OCD and ASD, *NOS1AP *may contribute to the disorder. Further genetic and functional studies are warranted to understand the role of *NOS1AP *in the susceptibility to psychiatric disorders.

## Competing interests

The authors declare that they have no competing interests.

## Authors' contributions

RD carried out molecular genetic studies for all the families, and drafted the manuscript. CB contributed to the collection of clinical data and DNA samples. IS, HA, PC, FS, GN, EH and MR identified and diagnosed the patients. MCM helped to collect the data. TB designed the study and revised the manuscript. ML and CG were principal investigators. All authors read and approved the final manuscript.

## Pre-publication history

The pre-publication history for this paper can be accessed here:

http://www.biomedcentral.com/1471-2350/11/108/prepub
